# Difficulties in daily life and the association with self‐care ability in adults with type 1 diabetes mellitus in Japan: A cross‐sectional study

**DOI:** 10.1002/nop2.466

**Published:** 2020-03-18

**Authors:** Eiko Umeda, Yasuko Shimizu, Nobuko Kawai

**Affiliations:** ^1^ Division of Health Science Osaka University Graduate School of Medicine Suita Japan

**Keywords:** difficulties in daily life, experience, self‐management, type 1 diabetes, worry

## Abstract

**Aim:**

To identify the unique contents of difficulties experienced in daily life among adult type 1 diabetes mellitus patients and to determine how basic characteristics and diabetic‐related self‐care agency are associated with difficulties in daily life.

**Methods:**

This study used two surveys: “Difficulties in Daily Life,” which was a questionnaire developed for this survey and “Instrument of Diabetes Self‐Care Agency.” These two measures were then used with demographic information for cross‐sectional analysis.

**Result:**

The final sample included 321 type 1 diabetes mellitus patients. Difficulties in daily life were composed of four factors: “Difficulties in diseases disclosure,” “Difficulties accommodating diabetes into daily life,” “Difficulties in employment or continuing education” and “Lack of knowledge of diabetes.” The instrument of Diabetes Self‐Care Agency was found to be associated with all four factors of difficulties in daily life. In addition, sex and diabetes duration were shown to be important predictors.

## 1 INTRODUCTION

Type 1 diabetes mellitus (T1DM) is a demanding disease that requires self‐management over a lifetime due to potentially serious complications. Approximately 95% of diabetes cases in Japan are type 2 diabetes mellitus (T2DM) (Patient Survey, 2015). The average annual incidence rate of T1DM in Japan is 1.4 to 2.2/100,000 people (Japan Diabetes Society, 2017; Kuzuya, Ito, Sasaki, Seino, & Tajima, 1992), which is very low. The age distribution at diagnosis (onset) of T1DM shows a peak at infancy and at age 10 to 13 (DIAMOND Project Group, 2006), and approximately 60% of these patients are female (Medical Aid for Specific Chronic Disease of Children, 2012). Therefore, there are more studies of children with T1DM than adults in Japan (Matsumoto & Hotta, 2017; Nakamura, Kanamaru, & Aya Nakai, Yuriko Kanematsu., 2016; Nakamura et al., 2017; Yamasaki & Tomari, 2016).

A previous study of adult T1DM found that adult T1DM requires self‐management behaviours to maintain the same social life as before being diagnosed with diabetes (Fisher et al., 2015). However, difficulties associated with the continuity of social life have been indicated (Fisher et al., 2015). There is a need to understand the difficulties associated with the delivery of care in daily life in adults with T1DM. Living with T1DM in adulthood is challenging because the many developmental and psychosocial demands of daily life compete with diabetes‐related self‐management tasks, thereby increasing the risk of psychosocial distress combined with poor glycemic control. Psychosocial distress is particularly common among young adults and has been connected with poor glycemic control. Those with a combination of poor glycemic control and psychosocial difficulties are especially prone to a poor prognosis (Balfe et al., 2013; Helgeson, Escobar, Siminerio, & Becker, 2010).

## 2 BACKGROUND

Adulthood is a developmental period marked by significant psychological and social changes. Adults face multiple challenges, including work, marriage, shifting relationships with family members, building a successful career and contributing to the local community. When proper glycemic control is not practiced during this developmental period, serious complications of diabetes can develop in late adulthood. Less than ideal glycemic control increases the risk for long‐term microvascular complication (Diabetes Control & Complications Trial Research Group, 1993). Health behaviours and diabetes self‐management behaviours formed in adulthood tend to persist later on, which is why support during this period is vital (Harris, 2010). Adult T1DM patients may face great difficulties with managing their T1DM when confronted with challenging life events. Life events that are unexpected may also occur during this period, such as job loss or death of a family member (Arnett, 2000). In young adults, problems at work and school are reported to be the most stressful life events (Hagger, Hendrieckx, Sturt, Skinner, & Speight, 2016; Joiner, Holland, & Grey, 2018). Diabetes distress in T1DM patients has been related to age at onset and sex (Lašaitė, Ostrauskas, Žalinkevičius, Jurvucieene, & Radzeviciene, 2016). However, no studies appear to examine what kind of life event‐related difficulties T1DM patients experience throughout adulthood.

The life events that patients with T1DM experience in adulthood are highly individual and varied. It is therefore becoming very difficult for nurses to understand what experiences patients with T1DM have in adulthood and in what aspects of these experiences patients face difficulties. Accordingly, there is a risk that the appropriate timing of nursing intervention and support is being missed. If nurses knew the contents of difficulties experienced by patients with adulthood T1DM in daily life and the factors that influence these difficulties, they could identify high‐risk patients and develop support plans at the appropriate time. The aim of this study was to identify the unique contents and frequency of difficulties experienced in daily life among adults with T1DM and to determine how sex, diabetes duration, A1C level, marital status and diabetic‐related self‐care agency is associated with difficulties in daily life. This information is expected to help identify patients with T1DM at risk of diabetic control.

## 3 METHODS

### 3.1 Study design

This was a descriptive cross‐sectional study using a self‐reported questionnaire.

### 3.2 Participants

We conducted this study in 2017–2018 at hospitals specializing in diabetes mellitus. We conducted an Internet search to identify diabetes clinics that perform examinations of patients with T1DM and sent a request to participate in the study to 27 institutions in Japan. Of these institutions, 17 agreed to participate. A total of 907 adult T1DM patients were randomly selected from these 17 institutions. The questionnaire was given to T1DM patients aged 20–65 years old by their doctor. A total of 379 patients (41.7%) agreed to participate and were recruited for the study. Of those that agreed to participate, 37 were excluded because they were outside the study's age range and 21 were excluded because they returned incomplete questionnaires. Therefore, the final sample included 321 T1DM patients. The questionnaire collected information on the following demographic variables: age, sex, age at T1DM onset, diabetes duration, employment status, marital status, insulin delivery method and A1C level.

### 3.3 Measurements

#### 3.3.1 Questionnaire on difficulties in daily life

We measured difficulties in daily life related to the experience of being diagnosed and living with T1DM. A literature review was conducted to identify themes related to difficulties in daily life related to T1DM, focusing on events and relationships specific to adulthood, including social relationships, work, marriage, parenting and leisure activities.

A search of the literature was done using the keywords “Diabetes Mellitus, Type 1” + “Self‐management or Self‐care” + “Patient Education” and “Diabetes Mellitus, Type 1” + “Life Change Events.” The result was 685 hits for papers published outside of Japan and 324 hits for papers published in Japan. Of these, 23 papers included descriptions of difficulties in the daily life of patients with T1DM. An item pool was created based on these 23 papers and questionnaire items were examined. After the literature review, items to include as contents of the questionnaire were carefully selected through discussion between the authors and experts in diabetes. Thereafter, a preliminary questionnaire survey was conducted on 15 patients with adulthood T1DM and amendments were made to the written expressions. This questionnaire on difficulties in daily life included 51 items. Responses were rated on a six‐point Likert scale from 1 (strongly disagree)–6 (strongly agree), with higher scores indicating more problems in daily life and lower scores indicating fewer problems. This questionnaire was created and modified for use in this survey.

#### 3.3.2 Instrument of Diabetes Self‐Care Agency

The Instrument of Diabetes Self‐Care Agency (IDSCA) is used to assess self‐management ability in patients with diabetes mellitus. This instrument has confirmed internal consistency and validity, with Cronbach's alpha of .936 and Pearson's correlation coefficient of .804 between the test and retest. It consists of 40 items, which constitute the following eight factors of diabetes self‐care agency: ability to self‐manage, motivation to self‐manage, monitoring ability, application or adjustment ability, ability to acquire knowledge, ability to make the most of the support available, stress‐coping ability and body self‐awareness. The IDSCA was developed in a previous study, and responses are given on a six‐point Likert scale ranging from 0 (strongly disagree)–5 (strongly agree). (Miyawaki et al., 2015; Waki et al., 2016). Scores were 0–200 points, obtaining a high score indicates that the self‐care agency of an individual with diabetes is good. Permission to use the instrument in the present study was granted by the original author. This scale has been tested for reliability and validity (Miyawaki et al., 2015).

### 3.4 Statistical analysis

Analyses were performed using JMP14.0 Pro software. To obtain an overview of the participants, basic characteristics were analysed by simple tabulation.

After simple tabulation of 51 items representing “difficulties experienced in daily life,” factor analysis was conducted on 40 items that all participants answered after excluding 11 items that only applied to some participants depending on their basic characteristics (such as items only applicable to people with parenting experience).

The principal factor method was used for factor extraction, and promax rotation was used as the rotation method because a correlation between factors was assumed. Items to be omitted were examined based on a factor loading of .40, and items were verified by an expert panel. The mean scores and standard deviations for the 40 questionnaire items were calculated to confirm the score distribution. A ceiling effect was seen in five items, and a floor effect was seen in six items; these 11 items were consequently excluded from the analysis. A factor analysis was done with the 29 remaining items to verify initial eigenvalues. Analysis was started with four factors based on the cumulative contribution rate and a scree plot. The factor analysis was repeated by conducting analyses using the principal factor method and promax rotation, confirming commonalities and omitting items based on the factor loading.

Each factor underwent multiple linear regression analysis as dependent variables. Multiple linear regression analyses were used to examine the relationships among sex, A1C level, diabetes duration, marital status, diabetic‐related self‐care agency and difficulties in daily life.

### 3.5 Ethical considerations

Participation in this study was voluntary, and participants were informed that they could withdraw without giving any reason. The procedures and protocols used in this study were approved by the Human Ethics Review Committee of Osaka University and by the ethics committees of the participating hospitals. This study was conducted in accordance with the Declaration of Helsinki, and written informed consent was obtained from all study participants.

## 4 RESULTS

The characteristics of the 321 participants are summarized in Table 1. The mean (standard deviation [*SD*]) age of all participants was 45.2 (10.9) years. Females comprised 67.0% of the participants. The mean duration of T1DM was 18.1 (11.1) years, and mean age at onset was 27.0 (14.2). Of all the study participants, 57.7% were working full‐time, 19.2% were working part‐time and 23.1% were unemployed. Multiple daily injections were used by 85.4%, and an insulin pump was used by 14.6%. Median A1C was 7.5 (1.1) %. The mean total IDSCA score was 133.2 (24.8).

**Table 1:** Characteristics of the study participants (*N* = 321)

Characteristic	%	*n*	Mean (*SD*)
Age (year)			45.2 (10.9)
Sex, *n* (%)			
Females	67.0	216	
Males	33.0	105	
Age at onset			27.0 (14.2)
Diabetes duration (year)			18.1 (11.1)
Self‐reported HbA1C (%)			7.5 (1.1)
Frequency of self‐monitoring blood glucose (time/day)			4.0 (2.9)
Complications, *n* (%)	17.5	56	
Living alone, *n* (%)	14.6	47	
Employment, *n* (%)			
Working full‐time	57.7	180	
Working part‐time	19.2	60	
Unemployed	23.1	72	
Marital status, *n* (%)			
Single or divorced	35.9	115	
Married	64.1	205	
Insulin delivery method, *n* (%)			
Insulin pump	14.6	45	
Multiple daily injections	85.4	272	
Mean total IDSCA score			133.2 (24.8)
Ability to acquire knowledge			19.4 (4.1)
Stress‐coping ability			16.1 (4.7)
Ability to make the most of the support available			13.4 (6.0)
Monitoring ability			16.9 (4.2)
Application of adjustment ability			16.7 (4.4)
Motivation of self‐management			18.2 (4.2)
Ability to self‐manage			15.2 (4.9)
Body self‐awareness			17.2 (3.9)

Abbreviations: IDSCA, Instrument of Diabetes Self‐Care Agency; SD, standard deviation.

### 4.1 Difficulties experienced in daily life

Of the 51 questionnaire items regarding difficulties in daily life, the highest scoring item was “The large financial burden of diabetes treatment is a problem,” with a mean (*SD*) score of 5.3 (1.2). The items with the second and third highest scores were “I experience difficulty with being ineligible for health insurance,” and “I dislike being mistaken for having lifestyle‐related T2DM by the people around me,” with mean scores (*SD*) of 4.9 (1.9) and 4.8 (1.5), respectively.

### 4.2 Exploration of the components of difficulties in daily life

The factor structure was verified using an exploratory factor analysis to show the level of coherence in items regarding difficulties in daily life. This resulted in the extraction of a total of four factors and 17 items (Table 2). The first of the four factors was named “Difficulties in diseases disclosure,” the second factor was named “Difficulties accommodating diabetes into daily life,” the third factor was named “Difficulties in employment or continuing education” and the fourth factor was named “Lack of knowledge of diabetes.” In this study, Cronbach's alpha was .884.

**Table 2:** Factor loadings matrix of difficulties in daily life

No	Item	1	2	3	4
1	I sometimes worry about whether or not to tell my friends that I have diabetes	**0.948**	−0.097	−0.119	0.066
2	I sometimes worry about how much to reveal about my diabetes to my friends	**0.932**	−0.073	−0.161	0.141
32	I have worried about how much to reveal about my diabetes to people at my workplace or school	**0.843**	0.106	0.122	−0.118
31	I sometimes worry about whether or not to tell people at my workplace or school about my diabetes	**0.809**	0.119	0.156	−0.137
6	I have worried about when to tell my partner (or intimate friends) that I have diabetes	**0.697**	0.023	0.072	0.012
9	I sometimes worry about how much to reveal about my diabetes to my partner/spouse's parents (family)	**0.617**	0.039	0.105	0.058
39	Paying attention to meals is difficult because I am focused on work (household chores) or school work	−0.010	**0.808**	−0.110	0.038
37	I sometimes experience difficulty with my inability to self‐manage my diabetes as intended because I prioritize work (household chores) or school	−0.015	**0.795**	0.025	−0.038
38	I sometimes experience difficulty with my inability to do work (household chores) or school work as intended because diabetes self‐management takes time	−0.052	**0.652**	0.142	0.103
35	I have difficulty with my inability to attend scheduled medical examinations because of work (household chores) or school	0.144	**0.641**	−0.121	−0.028
41	I sometimes experience difficulty with my inability to successfully manage my diabetes when my family members are ill	−0.023	**0.555**	0.117	0.094
40	I sometimes experience difficulty finding a new hospital when I move or continue my education	0.027	**0.442**	−0.005	0.024
28	I sometimes feel I cannot obtain a job (position) I want or attend the school I want to because of my diabetes	0.027	−0.023	**0.839**	0.041
27	I sometimes feel that finding employment or continuing education is difficult because of my diabetes	0.042	−0.010	**0.814**	0.030
21	I sometimes have difficulty ascertaining truth from error because of the overabundance of new information on diabetes	0.001	0.027	0.041	**0.737**
22	I sometimes experience difficulty because I do not really understand the complications of diabetes	0.053	0.021	0.015	**0.645**
20	I dislike explanations being left out because medical professionals assume I understand everything due to my long disease history	−0.002	0.146	0.036	**0.486**

Factor correlations	1	2	3	4
1	—	0.346	0.361	0.286
2		—	0.540	0.454
3			—	0.367
4				—

Bold values indicate factor loading >0.4.

### 4.3 Factors influencing difficulties in daily life

The factor analysis revealed four factors cited as difficulties experienced by adult T1DM patients in daily life. To clarify the factors that predict difficulties in daily life, multiple regression analyses were conducted with sex, HbA1C, diabetes duration, marital status and IDSCA score as explanatory variables. Explanatory variables were selected from the previous study and from a medical point of view and were selected from those that are important in relation to outcomes. Multicollinearity was also considered. Preliminary analyses found no violation of the assumption of normal distribution of residuals, multicollinearity and homoscedasticity. In the analysis with “Difficulties in diseases disclosure” as the dependent variable, the multiple regression model predicted significance with four variables [*F* (5,299) = 15.0, *p* < .001, adjusted *R* ^2^ = .23]: sex (β = 0.29, *p *≤ .0001), diabetes duration (β = 0.22, *p *≤ .0001), marital status (β = 0.16, *p* = .002) and IDSCA score (β = 0.29, *p *≤ .0001). Next, with “Difficulties accommodating diabetes into daily life” as the dependent variable, two variables were significant [*F* (5,299) = 12.6, *p* < .001, adjusted *R* ^2^ = .16]: HbA1C level (β = 0.16, *p* = .048) and IDSCA score (β = 0.37, *p *≤ .0001). With “Difficulties in employment or continuing education” as the dependent variable, three variables were significant [*F *(5,299) = 9.13, *p* < .001, adjusted *R* ^2^ = .12]: sex (β = 0.13, *p *≤ .016), diabetes duration (β = 0.25, *p *≤ .0001) and IDSCA score (β = 0.26, *p *≤ .0001). With “Lack of knowledge of diabetes” as the dependent variable, two variables were significant [*F* (5,299) = 15.0, *p* < .001, adjusted *R* ^2^ = .19]: diabetes duration (β = 0.10, *p* = .048) and IDSCA score (β = 0.44, *p *≤ .0001; Table 3).

**Table 3:** Regression analysis summary of variables predicted to be difficult experiences in daily life among adult T1DM patients (*N* = 321)

Predictor	*B*	*SE*	s*β*	*t*	*p*	Adjusted *R* ^2^
“Difficulties in disease disclosure” (items 1, 2, 32, 31, 6, 9)						
Sex	2.93	0.51	0.289	5.71	<.0001	.23
HbA1C level	−0.41	0.45	−0.049	−0.92	.357	
Diabetes duration	0.19	0.04	0.217	4.29	<.0001	
Marital status	−1.58	0.51	−0.160	−3.11	.002	
Mean total IDSCA score	−0.11	0.02	−0.287	−5.44	<.0001	
“Difficulties accommodating diabetes into daily life” (items 39, 37, 38, 35, 41, 40)						
Sex	0.55	0.39	0.075	1.42	.158	.16
HbA1C level	0.68	0.34	0.110	1.99	.048	
Diabetes duration	0.05	0.03	0.078	1.48	.140	
Marital status	0.51	0.38	0.071	1.32	.187	
Mean total IDSCA score	−0.10	0.02	−0.367	−6.65	<.0001	
“Difficulties in employment or continuing education” (items 28, 27)						
Sex	0.45	0.19	0.131	2.42	.016	.12
HbA1C level	−0.06	0.16	−0.021	−0.37	.715	
Diabetes duration	0.07	0.02	0.246	4.54	<.0001	
Marital status	0.01	0.18	0.004	0.07	.941	
Mean total IDSCA score	−0.03	0.01	−0.262	−4.64	<.0001	
“Lack of knowledge of diabetes” (items 21, 22, 20)						
Sex	−0.17	0.18	−0.049	−0.93	.352	.19
HbA1C level	−0.11	0.16	−0.038	−0.70	.484	
Diabetes duration	−0.03	0.02	−0.103	−1.99	.048	
Marital status	0.25	0.18	0.075	1.43	.153	
Mean total IDSCA score	−0.06	0.01	−0.436	−8.04	<.0001	

Abbreviation: IDSCA, Instrument of Diabetes Self‐Care Agency.

## 5 DISCUSSION

Experiences following a diagnosis of T1DM that presented the greatest difficulty in the daily life of adults were the large financial burden of diabetes treatment, being ineligible for life insurance and being mistaken for having T2DM. The dislike for being mistaken for having T2DM was consistent with the results of a study by Balfe et al. (2013). Patients with T1DM are discontent with their condition being interpreted as an illness arising from obesity, overeating and a disrupted lifestyle, which are characteristics associated with T2DM (Balfe et al., 2013). In Japan, the term “diabetes” is used to denote T2DM; awareness of the distinction between T1DM and T2DM therefore needs to be raised among many people, including those in the media.

As for the economic aspects, the financial burden of T1DM is a constant concern throughout a person's life considering T1DM is an illness that requires continued injections of insulin and measurements of blood sugar. Public financial support to avoid conflict between the financial burden of T1DM and continued treatment and/or the prevention of complications may be necessary.

When the factors that influence difficulties in the daily lives of T1DM patients were examined, the IDSCA score was found to be a predictor of all four factors of difficulties in daily life. This revealed that IDSCA score implies a causal relationship with changes in the feeling of difficulty in daily life. In other words, improving the IDSCA score may reduce many feelings of difficulty in daily life among adults with T1DM.

The first factor, “Difficulties in diseases disclosure,” was predicted by sex, diabetes duration, marital status and IDSCA score. In terms of sex, being male was most strongly associated with difficulties in diseases disclosure. Women with T1DM are reported to experience great diabetes‐related distress (Lašaitė et al., 2016), which is opposite to the finding in the present study. Several diabetes studies have found significant differences in interpersonal distress by sex, with females presenting more interpersonal distress than males (Chesla, Kwan, Chun, & Stryker, 2014; Chlebowy, Sula Hood, & LaJoie, 2013). Longer diabetes duration and being single were associated with higher difficulties in disease disclosure. Patients with longer duration of T1DM may need to obtain advice from a specialist in diabetes from onset to be able to properly explain their disease to others. More importantly, the patient's own decision regarding whether or not to tell those around them about their diabetes should be respected. However, our role as healthcare professionals is to improve society so that these patients can talk about their T1DM without hesitation.

The second factor, “Difficulties accommodating diabetes into daily life,” was predicted by A1C level and IDSCA score. Higher A1C level and lower IDSCA score were associated with difficulties accommodating diabetes into daily life. Accommodating diabetes into daily life was an important factor in determining A1C and was the key factor for prevention against worsening blood sugar control. It is important to understand patients' difficulties in daily life and to provide advice for self‐management according to lifestyle.

The third factor, “Difficulties in employment or continuing education,” was predicted by sex, diabetes duration and IDSCA score. Male sex, longer diabetes duration and lower IDSCA score were associated with higher difficulty in employment or continuing education. In a survey of workers with T1DM, those surveyed were reported to experience job discrimination and unwanted attention at their place of work because of their diabetes (Hakkarainen, Munir, Moilanen, Räsänen, & Hänninen, 2018). Furthermore, a survey of university students found that currently, 90% of students with T1DM do not contact university support services despite experiencing poor glycemic control and severe hypoglycaemia (Kellett et al., 2018). These difficulties are said to be associated with the lack of knowledge and low sensitivity with regard to T1DM of personnel at these patients' workplaces and schools. Medical professionals therefore need to create opportunities to actively impart knowledge of T1DM at workplaces and schools. Providing T1DM patients with support for self‐care tailored to their respective job type or school environment is also important.

The fourth factor, “Lack of knowledge of diabetes,” was predicted by IDSCA score and diabetes duration. Shorter diabetes duration and lower IDSCA score were associated with lack of knowledge of diabetes. Those with a longer duration of T2DM showed better performance in self‐management activities in a previous study (Heisler, Piette, Spencer, Kieffer, & Vijan, 2005). The increased experience with self‐care activities for those with longer diabetes duration may have increased their knowledge of diabetes. Medical professionals providing patients with information on T1DM and offering advice on difficulties of daily life with T1DM could be effective solutions to help patients improve self‐management. However, a previous study reported that diabetes knowledge and self‐management alone are insufficient to improve glycemic control (Bukhsh et al., 2019). The challenge is fully incorporating diabetes self‐care into daily life. Further exploration is warranted to understand what factors are associated with an increase A1C level in T1DM patients with greater difficulties in daily life.

## 6 CONCLUSION

This study revealed the difficulties experienced by adult patients with T1DM in daily life. Patients with T1DM felt great difficulty with their condition being mistaken for T2DM and the financial burden of diabetes. Furthermore, difficulties in daily life were associated with lower self‐care agency.

Healthcare providers should recognize the impact of self‐care agency and diabetes duration on T1DM patients. Approaches to disease disclosure and employment or continuing education should focus on sex‐specific interventions to improve difficulties in daily life among adult T1DM patients. The life events that patients with T1DM experience in adulthood are individual. Thus, when devising individualized interventions, more attention should be given to each patient's “life history” in terms of knowledge acquisition, the acceptance of diabetes and the origins of this knowledge and acceptance.

### 6.1 Relevance to clinical practice

The questionnaire on difficulties in daily life created in the present study may be a useful clinical tool to assess difficulties with T1DM in daily life that puts emphasis on the perspective of the individual patient. We found that self‐care agency is an important factor that changes the feeling of difficulty in daily life in adult T1DM patients. Ongoing efforts to help T1DM patients improve self‐care skills throughout the life‐course should be made.

### 6.2 Limitations

To our knowledge, this is the first study to investigate difficulties in the daily lives of adults with T1DM at the national level in Japan. However, this was a cross‐sectional study, and therefore, interpretation of causality is limited. The final sample included 321 patients with T1DM, and there were more females than males. The reason for this is thought to be that females in Japan have a higher incidence of T1DM than males. Moreover, all measures were self‐reported; consequently, recall bias may have affected the results, which can also be considered a study limitation.

## CONFLICT OF INTEREST

The authors have no conflicts of interest to disclose.

## AUTHOR CONTRIBUTIONS

EU, YS and NK contributed to the study concept and design. EU researched the survey data and drafted the manuscript. YS analysed the data, interpreted the results and reviewed the manuscript. YS and NK interpreted the results and reviewed the manuscript. All authors critically reviewed and revised the manuscript and approved the final version. All authors take responsibility for the integrity of the data and the accuracy of the data analysis.

## Supporting information

Supplementary File S1
